# A report of the Sixth Annual Meeting of the International Society for the Prevention of Tobacco Induced Diseases (ISPTID)

**DOI:** 10.1186/1617-9625-4-11

**Published:** 2008-12-17

**Authors:** Parimal Chowdhury, Maxim Dobretsov

**Affiliations:** 1 Department of Physiology and Biophysics, University of Arkansas for Medical Sciences, 4301 W Markham Street, Little Rock, AR 72205, USA; 2 Department of Anesthesiology, University of Arkansas for Medical Sciences, 4301 W Markham Street, Little Rock, AR 72205, USA

## Abstract

The Sixth meeting of the International Society for the Prevention of Tobacco Induced Diseases (ISPTiD) was held in Little Rock, Arkansas on November 2–4, 2007 and has brought together 140 participants, scientists and experts in this specialized field from 30 countries across the World. The central theme of the conference was the "Translational Approaches to the Prevention of Tobacco Induced Diseases". Discussions held during the three days meeting's sessions (including poster session and platform discussion) promoted a better understanding of the connection between tobacco use and associated medical and health consequences. The Sixth Annual meeting of ISPTiD served as another successful step toward decrease in the huge sociological and economical burden that the entire World is facing with this addiction. The proceedings of the meeting were published in the conference booklet, the ISPTiD global web site and Cancer Database abstract web site. Funds generated from this meeting helped in part to establish the society's Journal "Tobacco Induced Diseases "into the major scientific journal index PubMed database and BioMed Central. The meeting set the tone for next the Annual meeting in Kyoto, Japan for the year 2008 with the theme "Tobacco free future".

## Background

Tobacco-induced disease is a well recognized burden on human society. It is estimated that in Hong Kong 5750 people die annually from active smoking and an additional 1300 from passive smoking. In China, about 100 million of the 300 million males currently aged 0–30 will eventually die from tobacco-induced diseases. Although great strides have been made in USA to make many states smoke free, smoking-related diseases and deaths are still continuing at an alarming scale. Worldwide, every ten seconds, another person dies as a direct result of tobacco use. Tobacco-induced diseases are the greatest potentially preventable causes of death, resulting in 5 million deaths globally each year.

The International Society for the Prevention of Tobacco Induced Disease (ISPTID) was founded in 2000 by Dr. Ed Nelson (University of Essen, Germany) with recognition that combined international efforts of professionals and educators from different areas of basic sciences, medicine and dentistry will provide a balanced approach devoting an equal attention to development of prevention, cessation and medical treatment programs in the area of tobacco-induced diseases.

In accord with this mission, the Society's aims are 1) to foster scientific medical studies and/or any ethically and legally permissible efforts that lead to prevention of tobacco abuse, tobacco addiction, and tobacco use- or second hand smoke-induced or exacerbated diseases; 2) to facilitate communication and dissemination of knowledge among independent scientists involved in such studies; 3) to promote and foster public health, pre-school and school health education to enhance human knowledge about the actual hazards of tobacco use; 4) to devise and promote health educational plans and strategies that would help to prevent recruitment of children to nicotine addiction; 5) to achieve wide multidisciplinary dissemination of analyses and new research information on the biomedical aspects of harm induced by tobacco, and the effectiveness of clinical- and population-based interventions to achieve smoking cessation.

The ISPTID annual meeting is the major forum of the society aimed to provide a platform for the coordinated discussions by the Society members, and development of the Society's strategies with a participation and feed-back from any concerned public. The first meeting was held in Essen in 2002. Since then successful conferences have been held in Winnipeg, Manitoba, Canada (2003), Louisville, Kentucky, USA (2004), Athens, Greece (2005) and Hong Kong, China (2006).

The Sixth meeting of the society was held on November 2–4, 2007 in Little Rock, Arkansas, USA. As a continuation of the society's efforts toward development of multidisciplinary approaches, the focus of the planning and organizing committees of this last meeting was to appeal as much as possible to a broader range of clinicians, scientists and other tobacco-control advocates from many backgrounds and from all over the world.

Toward this goal the theme "Translational Approaches to the Prevention of Tobacco Induced Diseases" was selected for the 2007 ISPTID conference and special efforts were made to develop a challenging program devoting an equal attention to all scientific and clinical issues associated with prevention and treatment of tobacco addiction and tobacco-induced diseases. In addition to regular and plenary sessions, two workshops on the treatment of nicotine addiction, tobacco control and utilization of tobacco settlement funds in research and public education, were planned during the conference. As a result, the Sixth meeting of the International Society for the Prevention of Tobacco Induced Diseases (ISPTID) in Little Rock brought together the 140 participants, scientists and experts in this specialized field from 30 countries around the world. Discussions held during the 23 meeting sessions (including poster session and platform discussion) promoted a better understanding of the connection between tobacco use and associated medical and health consequences. The Annual meeting of ISPTID served as another successful step toward the huge sociological and economical burden that the entire world is facing with this addiction.

## The meeting organization and program focus points

The conference was organized with a premise that a successful meeting should provide a platform to foster collaborations and initiate new research while also providing training opportunities for interested graduate and post graduate students seeking to work in the field of tobacco addiction and associated health and sociological problems. Accordingly, there were two major focus areas: 1) to bring together US and foreign speakers and participants from multiple disciplines essential to the proper study of drug addiction (tobacco smoking) and associated health consequences. 2) To provide training experience for graduate students, postdoctoral fellows, and young investigators in the area of drug addiction (tobacco smoking) and health research. The third important goal was to establish a flawless conference program covering all associated scientific and logistical aspects starting from the first announcement of the conference through the final plenary session and panel discussion

Two very important committees were created and each one worked in close collaboration with the other. The first committee (Organizing Committee) was represented by both local and international members with expertise in scientific and organizational aspects including the selection of the theme of the conference. This committee met once a month. The second committee known as Logistics Committee was represented by scientific members in addition to other key people who were known for their organizational skills of a meeting of international caliber. This committee met once a month and more as required.

### Program, themes, speakers (program committee)

The first goal was formulated with the understanding that solution of the problem of drug addiction (tobacco smoking) and associated health consequences requires joined efforts of psychologists, sociologists, clinicians and experts from many different areas of clinical and basic sciences (including but not limited to cardiology, pulmonology, oncology, neurology and neuroscience, epidemiology etc). Consequently, the meeting program was designed around disciplines and grouped by those themes that are currently of greatest relevance in understanding the relationship between tobacco smoking and associated health problems. The program featured plenary lectures and discussion groups aimed at fostering the free flow of ideas and highlighting of problem areas in drug addiction that demand specific research attention. In addition, chairs of sessions (both plenary and scientific) have been carefully selected from National and International participants.

### Abstract selection and travel awards (abstract committee)

To meet the second goal (providing a learning experience in the field of drug addiction (tobacco smoking) and health research for young scientists), graduate students, postdoctoral fellows and junior investigators from the US as well as from other, and specifically underdeveloped countries were encouraged to submit abstracts and several steps were taken by the meeting organizers to provide the incentive and support for most distinguished applicants.

First, during the abstract review process all submitted abstracts were considered as potentially competing for the ISPTID 2007 travel awards. Unbiased character of the review process was achieved in the following way. Each submitted abstract was reviewed by two independent experts in the field, who were asked to score the abstract in four categories, suggest the most appropriate form and/or category of presentation, and provide a recommendation regarding the travel award. The total score (maximum of 20) given to the abstract by the reviewer was normalized to the median of all total scores given by this particular reviewer, and then an average of two normalized scores for each abstract was calculated and total distribution of all scores from all submitted abstracts was built, with a cut-off travel award score of above 1.0. The value of the stipend was determined considering the total amount of funds available. A $1200 amount of award was issued to the presenting authors of the abstract recommended for award by at least one of the reviewers. As an additional incentive, registration fees were waived for all travel awardees. A total 17 of ISPTID 2007 participants received awards and 15 awards were issued (two awardees could not attend the meeting due to unforeseen circumstances).

Furthermore the Ed Nelson Best student award served to facilitate the meeting attendance by students and to honor the Founder of the society. Continuous Medical Education (CME) credits were provided as incentive to clinicians in attendance. Furthermore, registration fees were waived or lowered for Arkansas Department of Health and selected Arkansas community attendees. It was also waived for students-helpers from UAMS providing for them an opportunity to participate in an International Meeting

### Logistics (planning and logistic committees)

The third goal (flawless running of the conference) was achieved because of closely coordinated work of committees and expertise of stuff of UAMS office of Continuing Medical Education and the Department of Physiology and Biophysics. A fully independent website was developed by one person in charge having no dependence on outside intervention resulting in fast updates of the meeting information. In addition two Organizing and Logistics Committees consisting of many national, international, local individuals worked together closely to for this successful meeting.

#### Funding

Many contributors and sponsors supported this meeting impartially for its intended cause. The meeting was supported by funds from National Institutes of Health which includes support from National institutes of Drug Abuse and National Cancer Institute. In addition, the meeting was funded by International Society for the study of lung cancer. The corporate funding was received from Pfizer, Inc and Filligent Inc. Without their help the hosting of this meting at this site would not have been possible. The sponsorship support helped to bring many scientists, graduate students and non-scientists all across the globe.

#### The meeting's statistics

The meeting was attended by 140 participants from 30 countries (Argentina, Austria, Bangladesh, Belgium, Canada, Central African Republic, China, Czech Republic, France, Greece, India, Iran, Iraq, Japan, Lithuania, Libya, Mexico, Nigeria, Pakistan, Poland, Romania, Russia, South Korea, Sweden, Taiwan, Thailand, Tunisia, Turkey, Uruguay, and United States of America); Forty eight participants are women. Representatives of disciplines ranging from medicine, basic science, nursing, jurisprudence to dental medicine joined in three days of the meeting. US Government agencies represented in Plenary and other sessions included Center for Disease Control and prevention (CDC), National Institutes of Health (NIH) including National Cancer Institute (NCI) and National Institutes of Drug Abuse (NIDA). State agencies represented in the meeting were the Arkansas State Government, Arkansas Department of Health, UAMS College of Public Health, and the State Health and Human Services. In addition, the University of Arkansas for Medical Sciences, Arkansas Children's Hospital, University of Arkansas at Fayetteville, University of Arkansas at Little Rock, University of Central Arkansas, University of Arkansas at Pine Bluff and the Arkansas State University were also represented at the meeting. Finally, the meeting provided an opportunity for the Canadian Author/Illustrator Yvonne Hedley to showcase her "The No Smoking" Experience Activity Book (health education for children ages 5–11) to the ISPTID 2007 Delegates from around the World.

There were a total of 8 plenary sessions, 2 workshops, 11 scientific sessions (with total of 56 oral talks presented and discussed) and a poster session with a total of 29 posters on display. The details of these sessions are described below:

##### Plenary Sessions

1. Addiction (Dorothy Hatsukami, U Minnesota) Chair: Dr. Jagjit Singh Khalsa, NIDA/NIH

2. Second Hand Smoke (Terry Pechacek, Center of Disease Control) Chair: Dr. Carolyn Dresler, AR Dept of Health and Human Services

3. Cancer and Prevention (Cathy Backinger, National cancer Institute) Chair: Dr. James Suen, UAMS, Dept of Otolaryngology, ACRC

4. Relapses in Smoking Cessation (Maxine Stitzer, John Hopkins University) Chair: Dr. Alan Budney, UAMS Dept Of Psychiatry

5. Behavioral Economics of Smoking (Warren Bickel, UAMS) Chair: Dr. Toshitoko Nakahara, Kyoto University, Japan

6. Smoking and Teen Pregnancy (Joycelyn Elders, Former Surgeon General of USA) Chair: Michael Jennings/Joseph Bates, UAMS/AR Department of Health

7. Clinical Epidemiology (Albert Lowenfels, NYU) Chair: Athanosios Zavras, Harvard University

8. Tobacco Control Policy Issues in Southeast Asia (Anthony Hedley, U Hong Kong) Chairs: Larry Suva, Orthopedics and Physiology, UAMS and Sarah McGee, University of Hong Kong

##### Scientific Sessions

1. Smoking and Cessation-Legal and Economical Issues – 1 Chairs: Yau, Sea-Wain, Taiwan and Satomura Kazunari, Japan

2. Clinical Management of Smoking Cessation Chairs: Taru Kinnunen, USA and Radahoune Fakhfak, Tunisia

3. Smoking and Cessation-Legal and Economical Issues – 2 Chairs: Dubois Gerard, France and Wheeler, J Gary, US

4. Toxicology: Cellular/Molecular Mechanisms – 1 Chairs: Scott, Elliott, Canada and Scott, David A, USA

5. Cessation and Prevention: Sociological and Psychological issues – 1 Chairs: Zavras, Athanasios, USA and Baroud, Thaer, USA

6. Toxicology: Cellular/Molecular Mechanisms-2 Chairs: Stites, Wesley, USA and Tang, Fusheng, USA

7. Cessation and Prevention: Sociological and Psychological issues – 2 Chairs: Ramstrom, Lars, Sweden and Sussman Steve, USA

8. Tobacco smoking related Disease – 1 Chairs: Chen George, Hong Kong and Reza, Hakkak, USA

9. Cessation and Prevention: Sociological and Psychological issues – 3 Chairs: Leen-Feldner, Ellen, USA and Charles R. Feild, USA

10. Tobacco smoking related Disease – 1 Chairs: Heulitt, Mark, USA and Bernhard David, Austria

11. Strategies for Industry Independent funding of research into Tobacco related Diseases Chairs: Chowdhury/Dobretsov: Presenters : Melissa Mowbray d'Arbela, Hong Kong and David Scott, USA

##### Workshops

1. Utilization of Tobacco Master Settlement Funds: The Arkansas Experience Moderator: Dr. Robert E. McGehee, PhD. Professor and Dean of UAMS Graduate School

2. Treating Tobacco Dependence in a Public Health Context: Overview of a Comprehensive Program Dr. Christine Sheffer, PhD. Assistant Professor, UAMS College of Public Health

## The meeting outcome

The Sixth meeting of the International Society for the Prevention of Tobacco Induced Diseases (ISPTID) in Little Rock has brought together 140 participants, scientists and experts in this specialized field from 30 countries around the world. Discussions held during the 23 meeting sessions (including poster session and platform discussion) promoted a better understanding of the connection between tobacco use and associated medical and health consequences. The annual meeting of ISPTID served as another successful step supporting the decrease in the huge sociological and economical burden that the entire World is facing with this addiction. The Meeting materials have been published in the Conference Booklet, the ISPTID web site and Cancer Database abstract web site. As an additional result, the meeting has raised funds that allowed inclusion of the Society's Journal, Tobacco Induced Diseases into the major scientific journal index PubMed database and BioMed Central.

Another important outcome of the meeting was the panel discussion that openly addressed the question about the ethical appropriateness of using funds provided by corporate businesses for tobacco research. The society made history on this issue since this is the first time the society ever planned such a discussion. As it could be anticipated, considering the delicate nature of the question, the opinions were sharply divided and no consensus was reached, except for organization of the ethics committee within the ISPTID. Nonetheless, the open discussion on whether scientific integrity and corporate sponsored funding are antagonistic entities, reverberated for some time after the meeting, and this should be considered as an important step forward for the society's future goals. It is anticipated that this discussion will continue and the balanced solution for the question will be achieved. In the opinion of the authors of this report, the success of the ISPTID 2007 Meeting is a clear example that corporate funding can be used without jeopardizing the integrity of aims and goals of the Society. Education of the general public in this respect could be an important part of general educational efforts of the Society.

## Conclusion and recommendations for future meetings

The Sixth Annual meeting of the ISPTID served as an important reminder to focus on the eradication of tobacco use, its addiction and resulting tobacco induced diseases. The meeting was successful with its focused goals. On a scale of 1 to 5, the CME survey responders gave highest scores to the selection of topics (3.76) but scored lowest (3.46) to the quality of oral presentations. By profession, the highest overall scores were given by social workers (3.92) and lowest overall scores were given by clinicians (3.37). Further emphasis on translational research in this important area is warranted. This conference can potentially serve as a continuing theme for future society meetings.

## Competing interests

The authors declare that they have no competing interests.

## Authors' contributions

PC and MD contributed equally in preparation of this manuscript. All authors read and approved the final manuscript.

